# Uncovering the Fungal Diversity and Biodeterioration Phenomenon on Archaeological Carvings of the Badami Cave Temples: A Microcosm Study

**DOI:** 10.3390/life14010028

**Published:** 2023-12-24

**Authors:** Shivankar Agrawal, Joshua Khumlianlal, Sarangthem Indira Devi

**Affiliations:** 1 Department of Phytochemistry, ICMR-National Institute of Traditional Medicine, Belagavi 590010, India; 2 Institute of Bioresources and Sustainable Development (Department of Biotechnology, Government of India), Imphal 795001, India

**Keywords:** biomineralization, carbonate solubilization, fungal ecology, high-performance liquid chromatography, organic acids

## Abstract

The Badami Caves are a significant example of ancient Indian rock-cut architecture, dating back to the 6th century. These caves are situated in the Malaprabha River valley and are part of the candidate UNESCO World Heritage Site known as the “Evolution of Temple Architecture—Aihole-Badami-Pattadakal”, which is considered to be the cradle of temple architecture in India. Our study aimed to investigate the diversity, distribution, and biodeterioration phenomena of the fungal communities present on the cave surfaces. The study also conducted a comprehensive analysis of fungal biodeterioration on the cave carvings. Utilizing specialized techniques, the dissolution of calcite, alterations in pH levels, and biomineralization capabilities of isolated fungal strains were monitored. Additionally, this study analyzed fungal acid production using high-performance liquid chromatography (HPLC). Our findings revealed that the major genera of fungi found on the cave surfaces included *Acremonium*, *Curvularia*, *Cladosporium*, *Penicillium*, and *Aspergillus*. These isolated fungi were observed to produce acids, leading to the dissolution of calcium carbonate and subsequent decrease in pH values. Notably, the dominant genus responsible for acid production and the promotion of biomineralization was *Aspergillus*. These discoveries provide valuable insight into the ecology and functions of fungi inhabiting stone surfaces, contributing to our understanding of how to preserve and protect sculptures from biodeterioration.

## 1. Introduction

Archaeological antiquities, encompassing architectural monuments, statues, tombstones, stelae, and sculptures made of stone, constitute a substantial portion of the world’s cultural heritage. These cultural artifacts often contain inorganic materials such as metals and stone, providing a conducive environment for microbial life [1]. The growth of microorganisms on these objects is influenced by various factors, including moisture, pH, light, temperature, and nutrients. The colonization of these artifacts by microorganisms is intricately linked to both the characteristics of the surrounding environment and the nature of the substrata [2]. Fungi, being chemoheterotrophic eukaryotic microorganisms, exhibit remarkable diversity, with up to five million species [3]. They play a crucial role in biotransformation and the biogeochemical cycling of elements in the biosphere, contributing to processes such as carbon, nitrogen, phosphorus, and metal cycling [4]. While material degradation is a natural process in nature, biodeterioration driven by microbes on historical objects can result in irreversible harm to cultural assets [5,6]. Fungi, in particular, are considered versatile biodeteriogens due to their ability to thrive in harsh environments like rocks and can induce biodeterioration through aesthetic, physical, and chemical impacts [7]. Fungal biodeterioration manifests in both biophysical and chemical forms [8]. Biophysical biodeterioration occurs through hyphae contraction and expansion, leading to alterations in the stone’s integrity. On the other hand, chemical biodeterioration involves the release of extracellular metabolites, acids, and metal-chelating compounds that can dissolve minerals, causing secondary biomineralization, such as fungal carbonate stone diagenesis, that replaces the original stone minerals [9,10,11,12]. Fungi also impact the aesthetic qualities of stone, influencing the formation of subaerial biofilms, contributing to chromatic shifts of inorganic pigments [13], and creating bio-pitting through the penetration of stone by dematiaceous fungi [14,15]. Under humid conditions, fungi like *Alternaria*, *Aureobasidium*, *Cladosporium*, *Epicoccum*, and *Phoma* create hyphal networks within the stone’s pores, settling on the stone from the air to perform fungal colonization [16]. The ability of fungal metabolites to induce calcite dissolution is well-established, but few studies have explored the potential harm to cultural heritage monuments [17,18,19]. Therefore, understanding fungal diversity, structural distribution, and action mechanisms is crucial for accurately diagnosing biodeterioration processes and developing safe and effective control techniques to preserve global monuments [20]. In 2015, UNESCO recognized the “Evolution of Temple Architecture—Aihole-Badami-Pattadakal” (Karnataka, India) as a World Heritage Site. Badami is renowned for its five rock-cut temples dating back to the sixth century, representing early Dravidian temple architecture. The temples feature ornate carvings that depict a fusion of Hindu, Jain, and Buddhist iconography, reflecting the evolution of religious views over time. The construction utilized the sandstone and limestone escarpment of the hill for the Badami Cave temples and monuments [21]. Unfortunately, some structures in these caves exhibit signs of various biodeterioration processes, including significant bio-pitting observed on carvings. Many carvings in the caves show loss of outer layers due to these “fungoid” growths, indicating a microbiological origin. Surprisingly, there are no existing studies on the biodeterioration of carvings in the Badami Cave temples. In this study, two representative temples, Temple 1 dedicated to the Hindu God Siva and Temple 2 dedicated to Lord Visnu, were selected to explore bio-pitting and biodeterioration of carvings. Both temples are open to visitors and feature substantial carvings. 

The primary objectives of this study were threefold: (1) to investigate the structure and diversity of the fungal community, (2) to explore potential fungal sources of bio-pitting, and (3) to analyze the physiological characteristics of isolated fungal strains, including their biodegradation capabilities, organic acid production, and biomineralization potential. These findings carry significant value in advancing preservation efforts for the historic carvings within the Badami Caves.

## 2. Material and Methods

The used methodologies in this interdisciplinary study were carried out within two primary research areas: (1) biological, with the goal of fungus isolation, identification, and biodeterioration tests; and (2) analytical, in order to characterize organic acid production by fungi using HPLC.

### 2.1. Description of the Studied Stone Carvings

**Figure 1:**
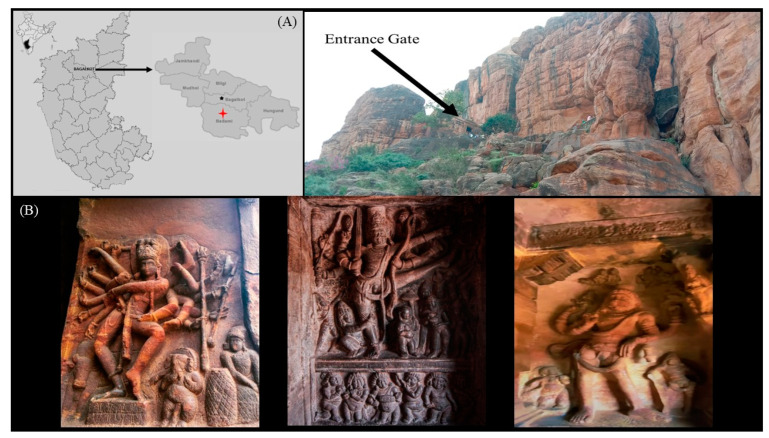
The Badami Cave temples sampled in this study. (**A**) Geographical location; (**B**) archaeological carvings.

Sampling was carried out with the necessary authorization from the Archaeological Survey of India in December 2021. The Badami Cave temples are situated in the town of Badami, located in the north-central region of Karnataka, India (coordinates: 15°55′06″ N 75°41′3″ E). These temples are approximately 88 miles (142 km) east of Belagavi and approximately 3 miles (4.8 km) from the Malaprabha River. The Badami Cave complex forms a part of the candidate UNESCO-designated World Heritage Site known as the “Evolution of Temple Architecture—Aihole-Badami-Pattadakal” in the Malaprabha River valley, recognized as the birthplace of temple architecture. Sampling was performed by gently scraping biofilm and dust material from stone carvings using sterile cotton swabs soaked in physiological saline solution (0.85% NaCl). Each carving was sampled in triplicate, and multiple heights on each carving were sampled. The collected samples were placed in 50 mL sterile Falcon tubes and stored at 4 °C during transport to the laboratory and until they were used for fungal culturing (see Figure 1 for details).

### 2.2. Mycological Analyses

#### 2.2.1. Fungal Isolation

Various procedures were used to isolate fungi from collected samples. The streaking procedure was performed by streaking swabs on potato dextrose agar medium and Czapek Dox agar medium plates (in three replicates). The rinse procedure was executed by shaking collected cotton swabs for 20 min in 10 mL of sterile distilled water. The water was then serially diluted and spread on PDA and CZA media plates [22]. Placing swabs on medium was also performed, in which samples were placed on Petri dishes with solidified medium. Petri dishes were incubated at 25 ± 2 °C for 28 days. Each day, plates were examined carefully to observe any visual fungal growth. Emerging colonies of fungi on the plates were picked up and sub-cultured on fresh media (isolates were purified using the single spore method) for morphological and molecular identification.

#### 2.2.2. Identification of Culturable Fungi

##### Morphological Analysis

After performing macro- and microscopic examinations of the colonies that developed on PDA and CZA media, morphological study of the isolated fungi was carried out. The fungi were identified using the taxonomic literature and diagnostic keys [23,24,25,26,27,28,29,30,31,32]. Subsequently, the fungi were genetically analyzed to confirm the affiliation of the species.

##### Molecular Analysis

Using the procedure outlined by White et al. [33], fungal isolates were identified using molecular methods based on the examination of their nuclear ribosomal internal transcribed spacer (ITS) region in rDNA. From cultures of each fungal isolate, chromosomal DNA was extracted, and PCR was used to amplify the ITS1-5.8S-ITS4 region of rDNA. Using the primer pairs ITS1/ITS4 (5′-TCC GTA GGT GAA CCT GCG G-3′/5′-TCC TCC GCT TAT TGA TAT GC-3′) and ITS1F/ITS4B (5′-CTTGGTCATTTAGGAAGTAA-3′/5′-CAGGAGACTTGTACACGGTCCAG-3′), as described by White et al. [33], the ITS region of the fungi was amplified. The 25 µL PCR reaction mix contained 2.5 µL of 10× buffer, 1.5 µL of MgCl_2_ (25 mM), 0.5 µL of dNTP (10 mM), 1 µL of each primer (5 pm), 0.2 µL of Taq polymerase (2.5 U), 3 µL of DNA sample (5 g/mL), and 15.3 µL of sterile Milli-Q water. The following settings were used to conduct the PCR reaction in the Bio-Rad C1000 Touch^TM^ Thermal Cycler: denaturation for 5 min at 94 °C, 33 cycles of elongation (50 s at 95 °C, 50 s at 56 °C, 1 min at 72 °C), final extension for 10 min at 72 °C, followed by a final step at 4 °C. In order to establish that there was no contamination, negative controls were performed.

The finished products were examined on 1% agarose (Sigma-Aldrich, St. Louis, MO, USA), and UV images of the gels (BioRad, Chemi Doc, MP, Hercules, CA, USA) were captured to show how they appeared. Using the QIAquick PCR Purification Kit, the PCR product was purified before being sequenced and analyzed using Finch TV 1.4.0.32. After purification from the gels, the resulting amplicons were sequenced on both strands. Grouped isolates with 98–100% similarity in both rDNA sequences were given entry numbers and their DNA sequences were uploaded to GenBank. The BLASTN program was used to identify the species by comparing it to the NCBI GenBank database and BLAST hits were obtained utilizing a difference from the query of less than 0.5% as the criterion. MEGA7.0.18.33 software’s neighbor joining technique was used to create the phylogenetic tree [34]. Analyzing the evolutionary relationships among these species involved employing the neighbor joining method. Percentages representing the clustering of associated taxa in the bootstrap test, conducted with 1000 replicates, were displayed above the branches in the resulting phylogenetic tree. The tree was drawn to scale, with the branch lengths reflecting the evolutionary distances used in its construction. The analysis involved a total of 22 nucleotide sequences, and the codon positions considered included 1st, 2nd, 3rd, and noncoding positions.

### 2.3. Biodeterioration Assays

#### 2.3.1. Determination of Carbonate Solubilization

For the evaluation of calcite solubilization, CaCO_3_ glucose agar medium was prepared, comprising 5.0 g of calcium carbonate, 10.0 g of glucose, 15.0 g of agar, and 1 L of deionized water (with an initial unadjusted pH), using the procedure outlined by Savković et al. [18]. After sterilizing the medium at 115 °C for 15 min, it was poured into Petri dishes to create agar plates. All isolated fungal strains were introduced into the plates and maintained in an incubator at a consistent temperature of 25 ± 2 °C for a duration of 4 weeks. The presence of calcite solubilization was confirmed by the development of a clear zone around positive fungal isolates.

#### 2.3.2. pH Changes of Substrate

To investigate the impact of fungal growth on substrate pH, Czapek Dox minimal broth, a low-nutrient medium with an initial pH of 7.5, was utilized. Each fungal strain was introduced into a 250 mL Erlenmeyer flask containing 50 mL of broth. These flasks were placed in a shaker set at a consistent temperature of 25 ± 2 °C and agitated at a rate of 120 rpm per minute. After a 7-day incubation period, the pH change was measured using a pH meter (Sartorius PB-30/PB-30L, One Science, Singapore) on the filtered supernatant obtained from the fungal liquid culture. This experiment was carried out in triplicate for accuracy and reliability.

#### 2.3.3. Organic Acid Analysis

To analyze the production of organic acids, a modified HPLC (high-performance liquid chromatography) method was employed, following the procedures described in previous studies [35,36]. Only those culture broths that exhibited a significant decrease in pH were subjected to analysis. Fungal liquid cultures underwent centrifugation at 12,000 rpm for 10 min, and the resulting supernatant was filtered through a 0.22 μm nitrocellulose filter. The HPLC system utilized was the Alliance system from Waters (Hawthorne, CA, USA). Analytes were separated via gradient elution on a Waters ODS2 HPLC analytical column (4.6 × 250 mm, 5 μm), maintained at a constant temperature of 25 °C throughout the experiments. The mobile phase was composed of solvent A (98% KH_2_PO_4_, 0.02 M, pH 2.8 ± 2 with o-phosphoric acid) and solvent B (3% methanol), with a controlled flow rate of 0.6 mL min^−1^. Organic acids were detected at a wavelength of 230 nm, and 20 μL of both standards and samples were injected for analysis, with each sample being analyzed in triplicate. To identify the types of acids present, HPLC peaks were compared with external standards, including oxalic acid, pyruvic acid, malic acid, lactic acid, acetic acid, citric acid, succinic acid, and fumaric acid, based on retention times and spectral data. As a control, a liquid culture without fungi was simultaneously examined for the presence of organic acids using the same method.

## 3. Results

In this study, an analysis was conducted of the fungal communities within nine samples taken from three distinct biodeterioration scenarios observed in archaeological carvings located in the Badami Caves, Karnataka, India. To achieve this, two distinct approaches were employed. The first approach involved culture-dependent DNA sequencing, while the second approach encompassed the utilization of analytical methods to complement the results. This dual method approach was adopted to address the limitations of each technique and to provide a comprehensive understanding of the fungal communities in the samples.

### 3.1. Morphology and Molecular Diversity of Culturable Fungi

**Figure 2:**
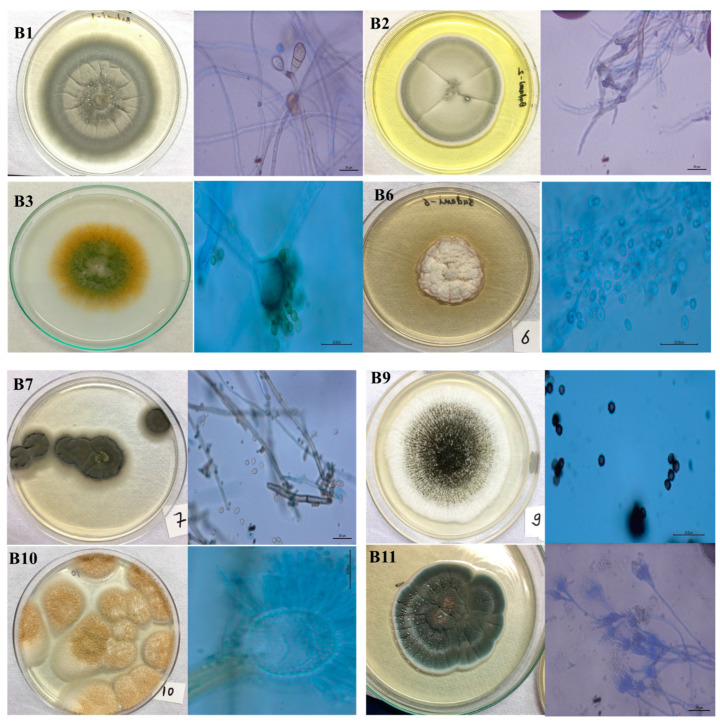
The morphological and microscopic appearance of the culturable fungi observed in the study: (**B1**) *Curvularia lunata*; (**B2**) *Cladosporium* sp.; (**B3**) *Aspergillus flavus*; (**B6**) *Acremonium persicinum*; (**B7**) *Cladosporium cladosporioides*; (**B9**) *Aspergillus niger*; (**B10**) *Aspergillus terreus*; (**B11**) *Penicillium chrysogenum*.

Conducting a mycological investigation on three carvings from the Badami Cave temples revealed the identification of various fungal strains. Each strain displayed diverse characteristics, including morphology, color, hyphal appearance, and secretion products. Figure 2 shows eight fungal isolates from the study, representing five different phyla.

*Curvularia lunata*: The colony border exhibited fluctuations between regular and irregular shapes during its development. There was a prevalence of a greyish colony and dark pigmentation was observed on the reverse side of the colony. The mycelia were septate. After an incubation period of 10 days, the presence of dark brown conidia, typically curved or sigmoid in shape, was noted. The conidia exhibited varied sizes but generally fell within the range of 20–40 μm in diameter.

*Acremonium persicinum*: The colony exhibited a whitish appearance, with the central region initially displaying a darker shade that gradually lightened as the colony expanded towards its periphery. The middle section took on a crumpled form during growth. The conidiophores were transparent, unbranched, and emerged individually either from a single vegetative hypha or from plectonematogenous hyphae.

*Cladosporium cladosporioides*: The colonies displayed an olive-colored with aerial mycelia that were diffuse and had a floccose-felty texture. The reverse side exhibited an olive-black color and was generally septate. The conidiophores were straight, solitary, unbranched, and could be found either at the terminal or lateral positions without nodules. Abundant conidia were present, showcasing a limoniform, ovoid, or obovoid to subglobose shape. They were aseptate, light brown, with prominent hila.

*Aspergillus flavus*: The mycelium exhibited a white or light-colored hue. Within a span of 3 days, the isolate generated conidia in shades of olive and dark green, ultimately assuming dominance in the colony aesthetics. These conidia, characterized by their small, round, or elliptical shape, formed chains. The conidiophores, lacking coloration, featured a thick wall with coarse roughening or pitting and were vesicle-bearing. Their diameter varied between 800 and 1200 μm.

*Aspergillus niger*: The mycelia were typically white or cream-colored. The conidia heads displayed a biseriate arrangement and exhibited a globose shape, featuring a broad spherical to globose vesicle measuring 37–52 µm. The stipe, characterized by a smooth texture, displayed a subtle brown hue. The conidia themselves demonstrated variability in size, ranging from 4 to 6 µm, and possessed a rough texture, appearing globose and brown. A distinctive diagnostic feature was the presence of a large and wide stipe.

*Aspergillus terreus*: The surface color ranged from pinkish cinnamon in early stages to a deeper hue with age, while the margins remained entirely smooth. On the reverse side of the colony, a spectrum of colors from pale yellow to bright yellow and deep brown was noted. The elevations exhibited an umbonate structure. The hyphae displayed branched septation, and the conidiophores were globose. The conidia were typically smooth and ranged in shape from spherical to ellipsoidal. Phialides were arranged in two series (biseriate), covering only the upper portion of the vesicle. Cleistothecia were absent.

*Penicillium chrysogenum*: The colonies displayed a distinctive blue-green hue. The conidia appeared smooth and globose, measuring 3 μm. The phialides took on a cylindrical shape, while the conidiophores were arranged in a bi-, ter-, and quarter-verticillate manner.

**Figure 3:**
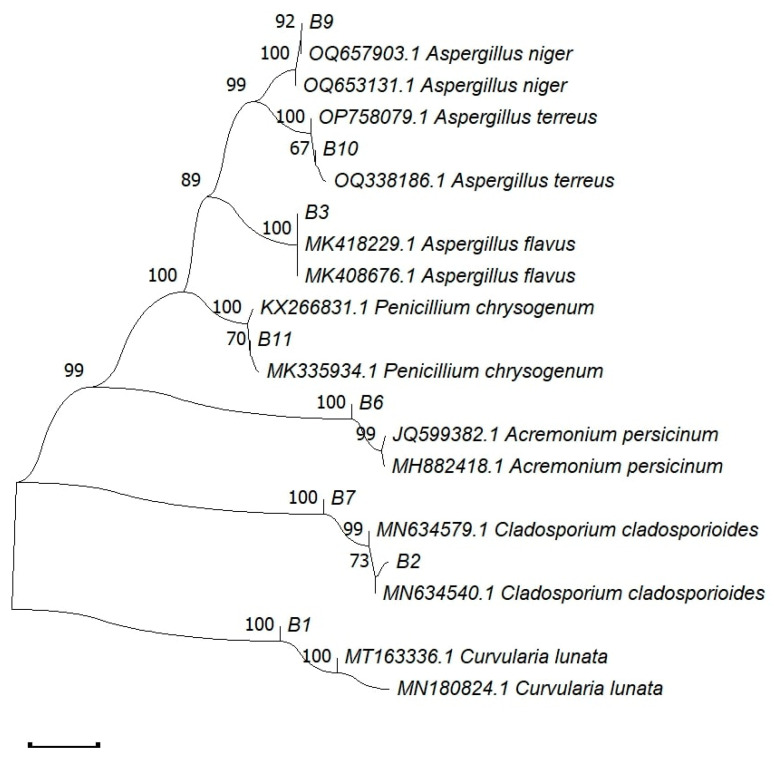
The phylogenetic tree representing fungi isolated from carvings of the Badami Cave temples.

Our BLAST analysis indicated that, except for strain B2, all cultures shared over 97% sequence similarity with fungal sequences in the GenBank database. These sequences were further classified into seven distinct species, primarily belonging to the Ascomycota phylum. The identified species include *Curvularia lunata*, *Cladosporium* sp., *Aspergillus flavus*, *Acremonium persicinum*, *Cladosporium cladosporioides*, *Aspergillus niger*, *Aspergillus terreus*, and *Penicillium chrysogenum*. The gene sequences have been deposited in the GenBank of the National Center for Biotechnology Information (NCBI) under the accession numbers OQ874797 to OQ874804, as shown in Figure 3 and Table 1. All of these fungal strains have been deposited in ICMR-NITM Microbial Repository. 

### 3.2. Biodeterioration Potential of Fungal Isolates

**Figure 4:**
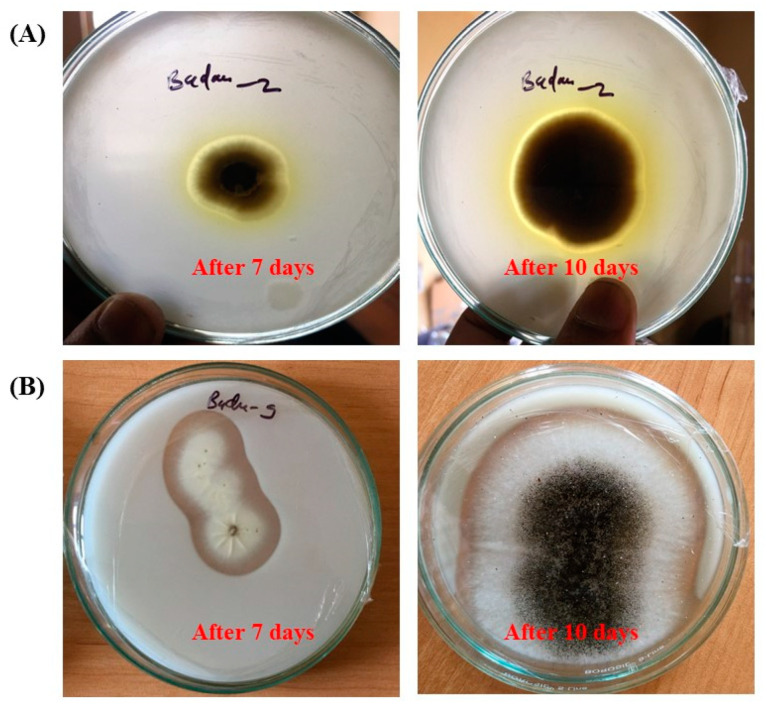
Calcite dissolution induced by fungal growth after 7 and 10 days of culturing and incubation. (**A**) *Cladosporium* sp., (B2); (**B**) *Aspergillus niger* (B9).

Figure 4 presents the results obtained from our biodegradation plate experiments. It was observed that the majority of cultivable fungi lacked the ability to dissolve calcium carbonate due to differences in their metabolic capabilities and ecological roles. Fungi have evolved diverse strategies for nutrient acquisition, and not all fungi possess the enzymatic machinery required to break down minerals like calcium carbonate (CaCO_3_). Only two isolates, namely B2 (identified as *Cladosporium angustisporum*) and B9 (identified as *Aspergillus niger*), displayed the formation of translucent clearing zones around their colonies, signifying their CaCO_3_-dissolving capabilities.

### 3.3. Changes in pH of the Liquid Medium

**Figure 5:**
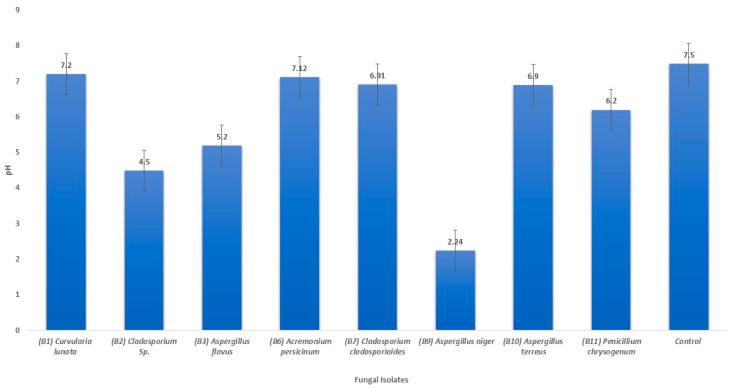
Changes in the pH value after 7 days of incubation in the oligotrophic broth medium.

Following a 7-day incubation period in pH 7.5 oligotrophic broth medium, a notable change in pH levels was observed, ranging from 2.24 ± 0.24 to 7.06 ± 0.14. In comparison to the control, all isolates exhibited lower pH values, as depicted in Figure 5. Notably, the culture medium of *Aspergillus niger* (B9) displayed the most significant pH alteration, decreasing from the control value of 7.5 to 2.24 ± 0.24. The pH reduction observed for the other fungal strains was likely the result of their limited production of organic acids.

### 3.4. Production of Organic Acids

**Figure 6:**
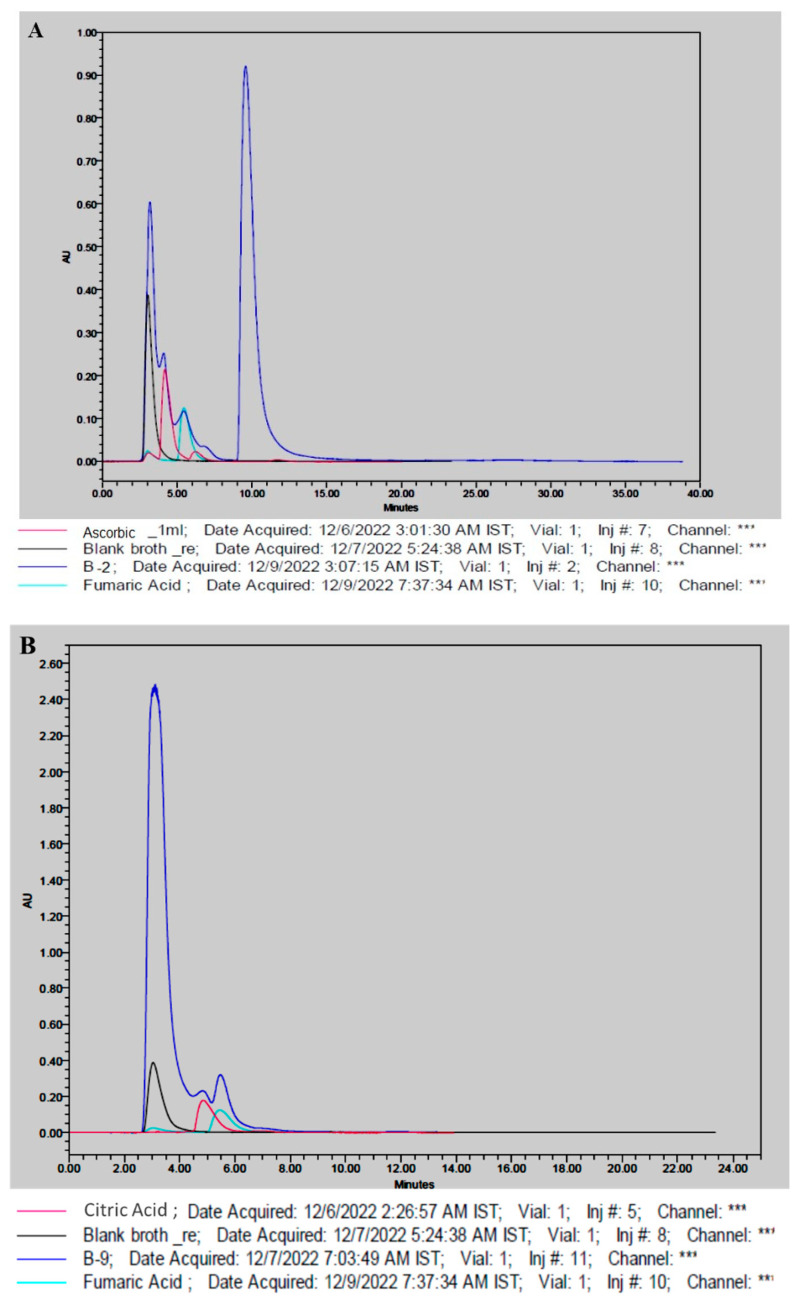
Overlayed HPLC chromatograms of blank, standard organic acid, and fungal culture media (**A**) *Cladosporium* sp., (B2); (**B**) *Aspergillus niger* (B9).

Using high-performance liquid chromatography (HPLC), the ability of fungal isolates B2 and B9 to secrete organic acids was examined. The analysis revealed several overlapping HPLC chromatogram peaks, indicating the presence of organic acids in the liquid medium when compared to standard organic acids. Figure 6 provides evidence of the production of ascorbic acid and fumaric acid by *Cladosporium* sp. and citric acid and fumaric acid by *Aspergillus niger*. The recorded retention times for the identified acids were 4.1, 4.5, and 5.4 for ascorbic acid, citric acid, and fumaric acid, respectively.

## 4. Discussion

Distinguishing between non-biological weathering and microbial-induced degradation of stone can be challenging, but it is undeniable that microorganisms play a role in stone deterioration. Urban areas, in particular, face significant degradation of various rock types on building facades due to atmospheric pollution. This degradation is attributed to chemical, physical, and biological processes. Rocks in urban environments tend to interact with and absorb pollutants, and the presence of microbial cells on stone surfaces can amplify the deleterious effects of pollution. Microbial cells may utilize deposited pollutants for growth, pigment production, and generation of corrosive metabolites, including acids [37,38].

The prevailing literature highlights the damage caused by acidic microbial metabolites as a primary factor in the degradation of constructed stone [39,40]. The concept of acid attack was initially proposed by Munz in 1890, attributing stone erosion to nitric acid produced by nitrifying bacteria [41]. This form of attack is often associated with the formation of pits on the stone surface. Fungi, on the other hand, are suggested to primarily degrade stone through the production of organic acids [9]. For instance, fungi isolated from deteriorated limestone in the Mayan site of Uxmal, Mexico, were found to produce oxalic acid, which reacted with solubilized calcium from the stone, resulting in the formation of crystals [42].

While organic acids are implicated in the degradation of stone, particularly limestone, through chelation, it is improbable that they directly degrade siliceous stone via acid attack. Instead, they likely act as chelating agents, enhancing cation solubility. Most minerals exhibit limited solubility in pure water, reaching equilibrium levels before significant lattice damage occurs. Organic chelating agents, such as acids and polysaccharides, increase cation solubility by reacting with released ions, forming soluble organic complexes that can permeate the stone’s pores and precipitate upon contact with a suitable substance, such as oxygen [43].

Research on the ruins of Argentine missions revealed evidence of iron and manganese mobilization from sandstone structures, with the surface layer enriched in these minerals compared to the interior. A complex biofilm on these buildings, containing various components, was identified as potentially contributing to mobilization and redeposition activities through cation chelation and acid production [44]. Bacteria [45], algae [46], cyanobacteria [39], and fungi [47] are known to produce a diverse array of chelating agents, including siderophores, which facilitate iron solubilization, uptake by cells, and potential transport and reprecipitation in other areas within the stone structure [48,49].

This research underscores the significance of employing a multidisciplinary methodology to investigate the complex phenomenon of stone artifact deterioration. Our results are consistent with earlier studies. In our investigation, the prevalence of specific fungal genera was noted—namely, *Alternaria*, *Aspergillus*, *Cladosporium*, and *Penicillium*—which were previously detected in Ançã limestone through metabarcoding analysis [19]. Furthermore, our results corroborate the earlier study’s conclusion that the mycobiota associated with various biodeterioration types exhibit distinct taxonomic characteristics, highlighting the significance of microenvironmental conditions in shaping these communities within similar lithological contexts. An examination of the enzymatic profiles of fungi was conducted using hydrolysable substrates incorporated into culture media and test strips. These substrates contained components typically encountered in historic canvas paintings. The study revealed that the genus *Aureobasidium* exhibited the highest level of biodeterioration potential, with *Cladosporium*, *Penicillium*, *Trichoderma*, and *Aspergillus* following in decreasing order of potential impact [50]. The formation of acid metabolites and solubilization of calcite in the case of fungal isolates indicated probable degradation. According to our findings, the majority of the fungal isolates belonged to the genera *Aspergillus* and *Cladosporium*, both of which had a clear tendency to dissolve minerals. Using CaCO_3_ glucose agar to isolate fungi from interior artworks, mural paintings, stone monuments, and ambient air, the researchers in [51] came to the conclusion that many species of the genera *Aspergillus* and *Penicillium* dissolve calcite. Numerous comparable investigations using artefacts from cultural heritage have demonstrated that a variety of *Aspergillus*, *Cladosporium*, and *Penicillium* species can produce considerable amounts of acids [4,52]. In a fascinating exploration, a study focused on ancient Chinese tombs dating back more than 1700 years unveiled intriguing insight into the differences in fungal community diversity between two tombs. These variations were likely influenced by differences in interior temperature, relative humidity, historical factors, and drawing techniques employed. Notably, *Penicillium aurantiogriseum*, *Aspergillus versicolor*, and *Penicillium olsonii* were identified in both tombs, comprising the core microflora responsible for the formation of black spots. These three fungi were presumed to be the primary sources of these dark markings. Over 68% of the isolated fungi exhibited proteolytic activity, while 27% of the strains produced acids, leading to the dissolution of calcium carbonate and a decrease in pH levels. Among the isolates, five out of six acid-forming fungal strains were also found to promote biomineralization. The genus *Penicillium* was the primary contributor to acid formation and biomineralization. These fungi, characterized by their biodeterioration and biomineralization capabilities, were abundant in the black spots, strongly suggesting their role in the formation of these distinctive marks. Exploring the production of fungal acids through additional research may uncover the microbiological and biochemical origins of decay. These organic acids play a pivotal role in the chemical deterioration of construction materials, concurrently reducing pH levels to foster an environment conducive to fungal proliferation, as opposed to bacterial growth [53]. The breakdown of calcium carbonate and pH value changes were employed in acid-production experiments, and HPLC was used to identify organic acids. The ability of certain *Aspergillus* and *Cladosporium* species to release fumaric acid is well recognized [54,55,56]. The findings indicate that archaeological monuments are at significant risk from flourishing fungi, primarily influenced by the specific characteristics of their local microenvironment. This study not only contributes to the scientific understanding of microbial communities on archaeological artifacts but also provides practical applications for the preservation and management of cultural heritage sites worldwide. The present study marks a groundbreaking venture in Indian archaeology, being the first of its kind conducted at a UNESCO World Heritage Site. This pioneering work not only contributes to our understanding of the region’s rich cultural heritage but also introduces innovative methodologies that could revolutionize archaeological practices in India.

## 5. Conclusions

In summary, our exploration of the fungal communities inhabiting the surfaces of the Badami Caves has illuminated the complex ecological processes at play and their implications for the preservation of this invaluable heritage site. The primary objective of our investigation was to gain a comprehensive understanding of the diversity, distribution, ecological functions, and interaction patterns of fungal communities thriving on the cave surfaces. Additionally, we sought to analyze the extent of fungal biodeterioration affecting the intricate cave carvings. 

Our study utilized specialized techniques, including monitoring calcite dissolution, observing pH level changes, and assessing the biomineralization capabilities of isolated fungal strains. These approaches provided crucial insight into the mechanisms of biodeterioration. Our findings highlighted the significant role of major fungal genera, such as *Acremonium*, *Curvularia*, *Cladosporium*, *Penicillium*, and *Aspergillus*, in the dissolution of calcium carbonate, leading to a decline in pH values. The application of HPLC technology accurately detected acid production, revealing the production of three acids—ascorbic acid and fumaric acid by *Cladosporium* sp. and citric acid and fumaric acid by *Aspergillus niger*. The dominance of the genus *Aspergillus* in acid production emerged as being particularly noteworthy, with implications for biomineralization.

These discoveries are of paramount importance in the context of heritage conservation, emphasizing the need to understand the ecological dynamics of fungal communities inhabiting stone surfaces for effective preservation strategies. The acid-producing nature of these fungi poses a tangible threat to the structural integrity of the Badami Cave carvings, underscoring the urgency of implementing measures to mitigate biodeterioration. Notably, *Aspergillus*, identified as a key player in these processes, warrants special attention in conservation efforts. 

## Figures and Tables

**Table 1:** Phylogenetic affiliations of isolated fungi.

Strain	Morphological Identification	Molecular Identification and BLAST Similarity	Identity (%)	Gen Bank Accession Number
B1	*Curvularia* sp.;	*Curvularia lunata*; (MT163336.1)	100%	OQ874797.1
B2	*Cladosporium* sp.;	*Cladosporium* sp., (MN634540.1)	73%	OQ874798.1
B3	*Aspergillus* sp.;	*Aspergillus flavus*; (MK418229.1)	100%	OQ874799.1
B6	*Acremonium* sp.;	*Acremonium persicinum*; (JQ599382.1)	99%	OQ874800.1
B7	*Cladosporium* sp.;	*Cladosporium cladosporioides*; (MN634579.1)	99%	OQ874801.1
B9	*Aspergillus* sp.;	*Aspergillus niger*; (OQ653131.1)	100%	OQ874802.1
B10	*Aspergillus* sp.;	*Aspergillus terreus*; (OP758079.1)	100%	OQ874803.1
B11	*Penicillium* sp.;	*Penicillium chrysogenum*; (KX266831.1)	100%	OQ874804.1

## Data Availability

Data is contained within the article.
